# Robust within-session modulations of IAT scores may reveal novel dynamics of rapid change

**DOI:** 10.1038/s41598-023-43370-w

**Published:** 2023-09-27

**Authors:** Aaron Cochrane, William T. L. Cox, C. Shawn Green

**Affiliations:** 1 Department of Cognitive, Linguistic, and Psychological Sciences, Brown University, Providence, RI USA; 2 Faculty of Education and Psychological Sciences, University of Geneva, Geneva, Switzerland; 3 Department of Psychology, University of Wisconsin-Madison, Madison, WI USA; 4 Inequity Agents of Change, Madison, WI USA

**Keywords:** Human behaviour, Attention, Cognitive control

## Abstract

The Implicit Association Test (IAT) is employed in the domain of social psychology as a measure of implicit evaluation. Participants in this task complete blocks of trials where they are asked to respond to categories and attributes (e.g., types of faces and types of words). Reaction times in different blocks sharing certain response combinations are averaged and then subtracted from blocks with other response combinations and then normalized, the result of which is taken as a measure indicating implicit evaluation toward or away from the given categories. One assumption of this approach is stationarity of response time distributions, or at a minimum, that temporal dynamics in response times are not theoretically relevant. Here we test these assumptions, examine the extent to which response times change within the IAT blocks and, if so, how trajectories of change are meaningful in relation to external measures. Using multiple data sets we demonstrate within-session changes in IAT scores. Further, we demonstrate that dissociable components in the trajectories of IAT performance may be linked to theoretically distinct processes of cognitive biases as well as behaviors. The present work presents evidence that IAT performance changes within the task, while future work is needed to fully assess the implications of these temporal dynamics.

Humans interact with dynamic worlds in which thoughts and behaviors must constantly be adapted to the current contexts ^1^. Accordingly, the presence of experience-driven change is recognized at some level in theoretical accounts of nearly all psychological processes. While this fact is perhaps most strongly emphasized in domains that focus on mechanisms underlying particular types of behavioral change (e.g., learning, fatigue, adaptation, maturational developmental processes, etc.), the core idea of the brain as undergoing constant experience-dependent updating is ubiquitous. For example, many theories characterize reasonably basic and fundamental aspects of perception and attention as inferences involving integration of current external information with established prior knowledge^2–4^.

In sharp contrast to this dynamic perspective, however, many psychological processes are studied using tasks and analysis methods wherein data is aggregated across large numbers of trials to create a single metric such as overall accuracy, reaction time (RT), or another index of performance. In this way, these methods, whether overtly or tacitly, are necessarily implementing the assumption that the behaviors of interest are unchanging over time. This assumption of stationarity can be a significant issue, as it is often unclear what an aggregated measure, such as an average, of a strongly time-varying process might “mean” with respect to theory or practice.

Importantly, even in those cases where there is reason to believe that certain processes or constructs of interest are reasonably stable (at least throughout the timescale studied)^5–7^, there is typically far less reason to believe that performance on the tasks used to measure those processes is similarly static^8–10^. While full consideration of this issue is rare, insinuated nods in this direction are relatively frequent in many domains. For instance, the typically unspoken rationale for employing some number of unanalyzed “practice trials” (something that is common across a wide range of studies situated in essentially all areas of psychology where behavior is measured at the trial-by-trial level) is that performance early in the task is somehow meaningfully different from performance later in the task. Thus, the goal is to remove from consideration those early trials where performance is changing and only utilize trials where performance on the task at hand is “stable.” In contrast, we have previously argued that in-task performance dynamics, when present, should not be stripped from the data^9,11,12^, but should instead be modelled. Not only does this eliminate what are frequently arbitrary choices as to the number of trials to treat as “practice,” but more much importantly those dynamics frequently offer critical information about the broader processes under study^9,11^. In short, by trying to eliminate the temporal dynamics in behavioral data, one runs the risk of missing out on important inferences that can only be obtained by examining those dynamics.

In the present work we examine short-term monotonic dynamics in the context of implicit evaluation, as measured by the Implicit Association Test (IAT^13^). The IAT is a task that typically uses a subtractive measure indexing differences in response times (RT) between combinations of image and word stimuli. For example, in one common version of this task, researchers would contrast the average RT in a block of trials that are “compatible” with anti-Black racial bias from the average RT in a block of trials that are “incompatible” with anti-Black racial bias (noting that many other variations of the IAT exist, including those without a racial component). In “compatible” blocks, participants push Button A when they see either a Black face or a negative-valence word and Button B when they see either a White face or a positive-valence word. In “incompatible” blocks, participants push Button A when they see either a Black face or a positive-valence word and Button B when they see either a White face or a negative-valence word. In essence, differences in RTs are taken to reflect the ease with which one can mentally bind Black/White faces with positive/negative words. Because this type of analysis aggregates all trials within a block and then subtracts aggregated values across blocks, it inherently assumes that responses to the stimuli of the various categories (i.e., combination types) are generated via temporally stable distributions of RTs. In other words, the analysis inherently assumes that RTs on the very first trial of a block are drawn from the same distribution (and, by extension, the same psychological generative process) as those on the 40th trial of the block. In addition, certain recommendations of IAT scoring^14^ implicitly weight a block of early trials more than later trials, although to our knowledge the detailed timecourse of performance has not been analyzed in order to examine the underlying dynamics supporting such a scoring method.

There are many reasons to believe that such an assumption of stationarity may not be borne out due to processes such as learning, self-regulation, or habituation^1,15^. Indeed, modification of behavior is a core part of typical human social interactions (e.g., in contexts of possible prejudice^16–18^), and recent work suggests that some biases may in fact be modifiable over a short time-scale^16,19,20^. The assumption of RT stationarity is also challenged by work showing learning in tasks that are structurally similar to the IAT, such as the flanker task (an attention measure of response compatibility and early inspiration for the IAT and related measures^16,21–26^).

Only by retaining dynamics of performance in data analysis (i.e., how participants’ IAT RTs change during the task) may patterns of change be observed. For example, under an aggregation-based approach, larger difference scores are simply associated with “more bias.” In this case, a participant who starts the task with a substantial degree of bias, but then very quickly unlearns that bias (or learns to suppress it in the task context) would appear to be exactly equivalent to a participant who starts with a lower degree of bias, but then never unlearns it (e.g., see Fig. 1). Under a Bayesian learning perspective^27^ the former might correspond to a participant who starts the task with a high magnitude, but reasonably flat prior (i.e., starts with high bias, but isn’t particularly committed to that bias, and can easily adjust their behavior). The latter, meanwhile, might correspond to a participant who starts the task with a lower magnitude, but much more peaked prior (i.e., a lower initial bias, but is quite certain of that bias being correct). If researchers attempted to use the IAT to predict overtly biased behavior, the most standard approach would make identical predictions for the two participants. Conversely, under the more dynamic perspective, one might expect the latter participant to show more biased overt behavior than the former, since they are less flexible in adapting their behavior to new situations.

**Figure 1:**
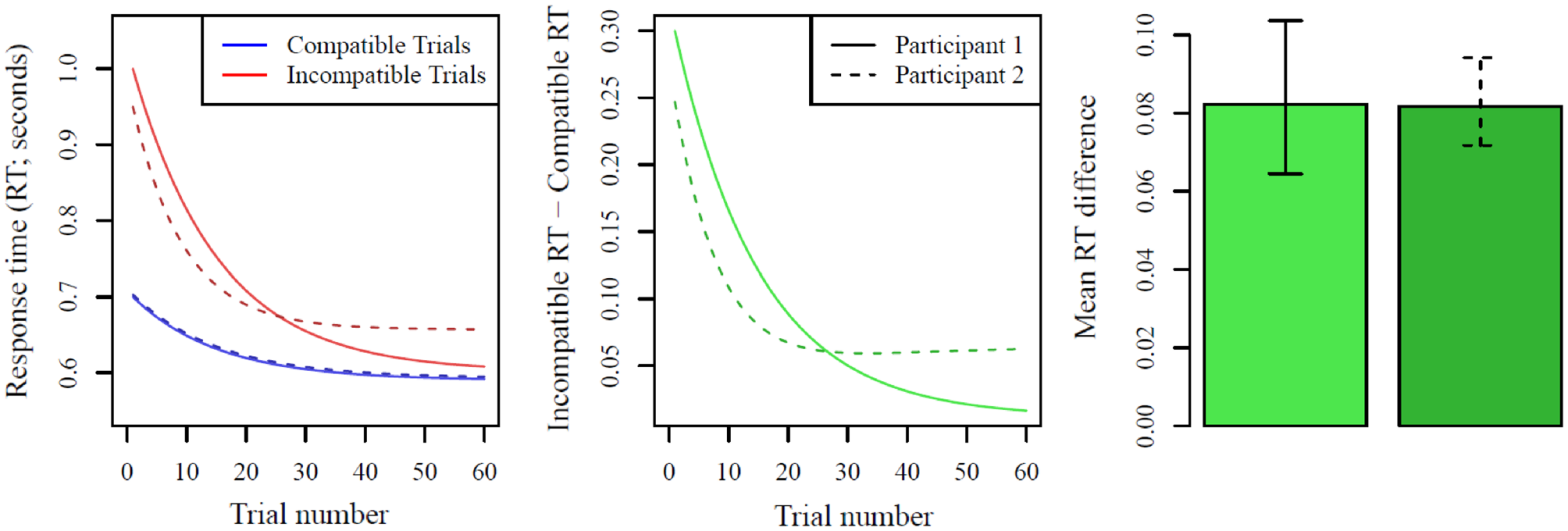
Two sets of artificial data generated to demonstrate how participants with the same IAT results (right panel), shown here as a mean RT difference between blocks (m_diff_ = .082 s) can have different trajectories of response times (left panel) and Block-type-related response time differences (center panel). Artificial participant 1 (solid lines) has a large initial difference between trial types, the two trial types change at the same rate, and by the end of the task the difference between trial types is very small. Artificial participant 2 (dashed lines) has a smaller initial difference than participant 1, response times for both trial types change very quickly but then level off at a position that results in a larger final difference than participant 1. Critically, because a standard mean-difference approach produces the same value for the participants, they would be expected to show the same level of bias in any follow-up or related measures. The dynamic approach meanwhile would not expect that the two participants would necessarily behave similarly in other tasks. Note that here we consider only the average difference in RTs and not the standard D score; similar points could be made using either aggregation approach.

While there has been a vibrant debate about whether the IAT has predictive validity for ecologically valid behaviors^13,28,29^, additional perspectives have questioned the use of the IAT as a singular predictor of real-world behavior without due consideration of other intervening processes, such as context-independent values, goals, and self-regulatory processes^16,30^. The present work can be seen as an extension of this re-calibration. In addition to other intervening processes that may interact with IAT metrics to predict behavior^16^, aggregation of IAT metrics may obfuscate or distort its links to more overt behaviors (e.g., in Fig. 1). That is, by identifying time-varying components of IAT change, such components could provide additional mechanistic specificity two possible ways: First, by providing a stronger signal regarding mechanisms of interest (e.g., persistent biases independent of learning), and second, by partialling out noise that would be included in an aggregation-based method.

In all, the motivating questions behind the series of analyses presented here are threefold, with each question building upon the next. The first question is the extent to which performance on the IAT changes as a function of time (i.e., within a typical session). The second question is whether IAT performance is amenable to time-evolving analyses which can identify the contribution of various stimulus attributes. The third and final question is whether the dynamic approach to the IAT allows for a better understanding of links between IAT and other manipulations and measures, such as survey or behavioral measures (Study 3) or learning interventions (Study 4). Each of these goals is exploratory in nature and is intended to open new avenues of inquiry, but not to posit specific mechanistic claims.

## Overall IAT methods

Standard IAT collection methods were utilized for Studies 1–3 below. More specificity is provided in each study’s section. In general, the IAT involves a 2-alternative forced choice in which participants must rapidly choose to press a button associated with stimuli on a computer screen.

All IATs were implemented in a standardized format with 7 blocks:

1. Single-stimulus training to associate response buttons with stimulus categories (e.g., White faces or words associated with competence)
2. Single-stimulus training to associate response buttons with stimulus categories of the category not trained in block 1.
3. 20 trials of of dual-stimulus task pairing (e.g., Good + Black & Bad + White or Good + White & Bad + Black)
4. 40 trials of dual-stimulus task pairing
5. Single-stimulus training to associate response buttons with the face stimulus categories on the opposite keys from blocks 2–4. Presented results used 40 trials, although this number can vary across versions.
6. 20 trials of of dual-stimulus task pairing opposite of block 3 and 4
7. 40 trials of dual-stimulus task pairing identical to block 6

## Study 1

The goal of Study 1 was solely to address the first of the questions noted above – whether there is evidence that performance on the IAT changes as a function of time (i.e., across blocks within a typical session). Specifically, we sought to demonstrate within-session changes on the IAT even when using relatively conventional scoring methods. In later studies we will build on this outcome with novel analytical approaches that are meant to more fully and continuously capture the particular dynamics at hand. As noted above, the typical methods of analysis used in the field at least tacitly assume that there are no such changes in performance (i.e., by using aggregation that assumes independently and identically distributed data). As such, here in Study 1, we made use of a large open IAT dataset. This data was already aggregated by block, yet we were able to take advantage of the presence of smaller initial blocks (considered “practice” in the original IAT studies^14^) to provide a first rough assessment of temporal change. An affirmative answer to the most basic level question would then justify more detailed and novel analyses of the temporal dynamics and whether aspects of the dynamics are differentially predictive of various outcomes (e.g., in Studies 2 & 3).

### Method

#### Data Source

Aggregated data for racial bias IAT from 2005 was acquired from Project Implicit^31^, a research project that has collected measures of racial bias over the Internet. We chose to restrict our analyses to one year’s data to ease the computational burden of analyses, and because we have no reason to believe that the choice of year would systematically influence results.

Participants were first excluded for incomplete sessions. Next, any sessions for individual participants that had previously completed an IAT task were excluded. This left 130,799 participants. Next, participants (*n* = 37,627) were excluded for accuracy below 70% or mean response times above 1.5 s on at least one block of trials, leaving 93,172 participants in the final analysis. While variation outside these exclusion criteria may be of interest in some contexts, the current goal was to include only participants who were very likely to be putting a good-faith, first-time effort into the task.

The aggregated IAT data had 6 variables of interest: participant D-score corresponding to the overall canonical IAT effect, the order in which conditions were presented (i.e., Black + Good first or White + Good first), and the four response time means, for blocks 3, 4, 6, and 7. As explained above, block 3 is identical to block 4 but with fewer trials and was originally labeled as practice; likewise for block 6 in relation to block 7.

#### Analysis

Three simple comparisons allowed for questions regarding the time-dependence of IAT results. First, we assessed whether the RTs were systematically different between participants’ blocks of identical trial types (i.e., blocks 3 vs. 4 and 6 vs. 7). This analysis allowed for the detection of rough, but systematic alterations in response times with experience (e.g., due to learning). Next, we tested whether the IAT D-score was systematically changed by the order of condition presentation. The presence of an order effect would similarly be indicative of experience-dependence of individuals’ D-scores. Last, we assessed whether between-participant effect sizes of condition differences were systematically different between blocks 3, 4, 6, and 7. Each of these assess, in different ways, the extent to which IAT performance varies over time.

### Results

Within participants, overall response times decreased from block 3 to block 4, paired *t*(93,171) = −304.83, mean_difference_4-3_ = −186 ms, CI = [− 187.2, − 184.8], d_Cohen_ = −2, *p* < 0.0001, as well as from block 6 to block 7, paired *t*(93,171) = −197.54, mean_diff_7-6_ = −109.6 ms, CI_difference_7-6_ = [− 110.7, − 108.6], d_Cohen_ = −1.29, *p* < 0.0001. Effect sizes were large in both cases.

**Figure 2:**
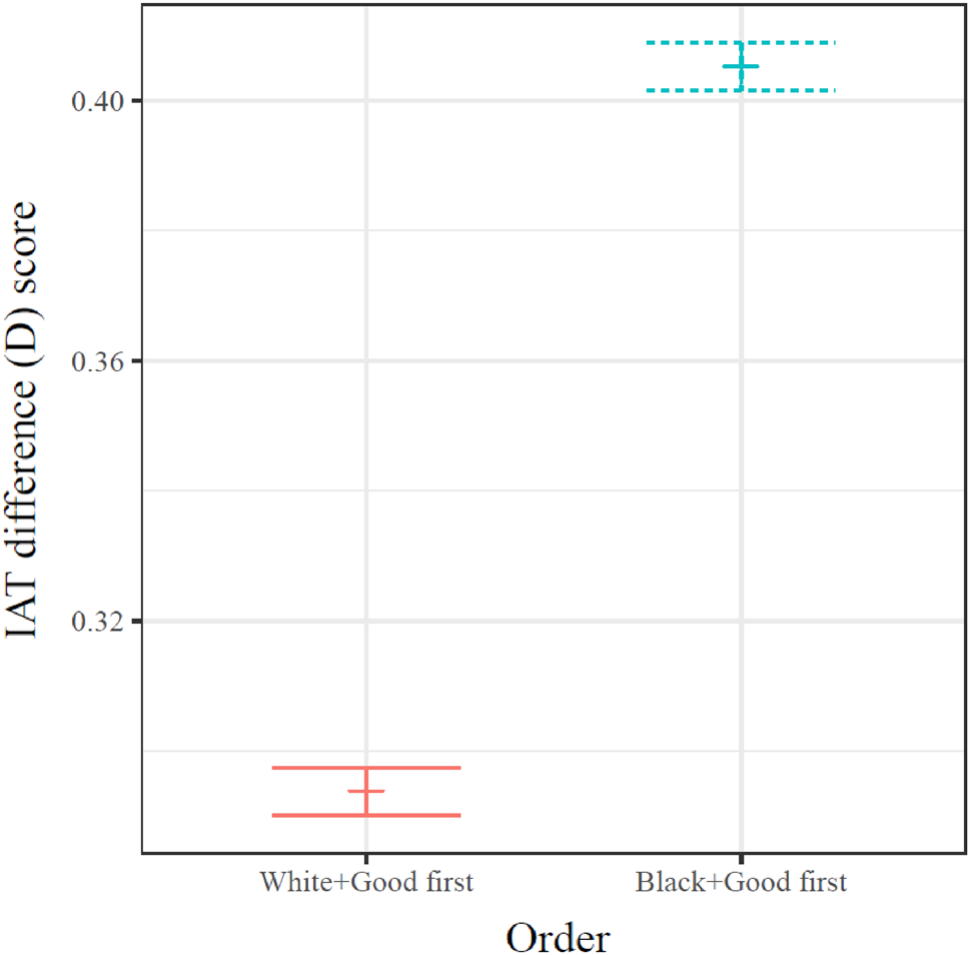
Comparison of IAT D scores of participants completing the Race IAT in each order. Participants completing the Black + Good Condition first systematically demonstrated larger IAT D scores. Central lines indicate means and error bars indicate bootstrapped 95% CI.

The data also indicated that the decreases in RT reported above were not merely task learning acting independently of trial types or inferences regarding IAT performance. Instead, an asymmetry was evident in within-trial dynamics; overall IAT D-scores were significantly related to the order in which the conditions were presented, Welch between-samples *t*(93,032) = −43.25, mean_compatible_first_ = 0.294, mean_compatible_second_ = 0.405, CI_difference_order_ = [− 0.116, − 0.106], d_Cohen_ = −0.28, *p* < 0.0001 (see Fig. 2).

**Figure 3:**
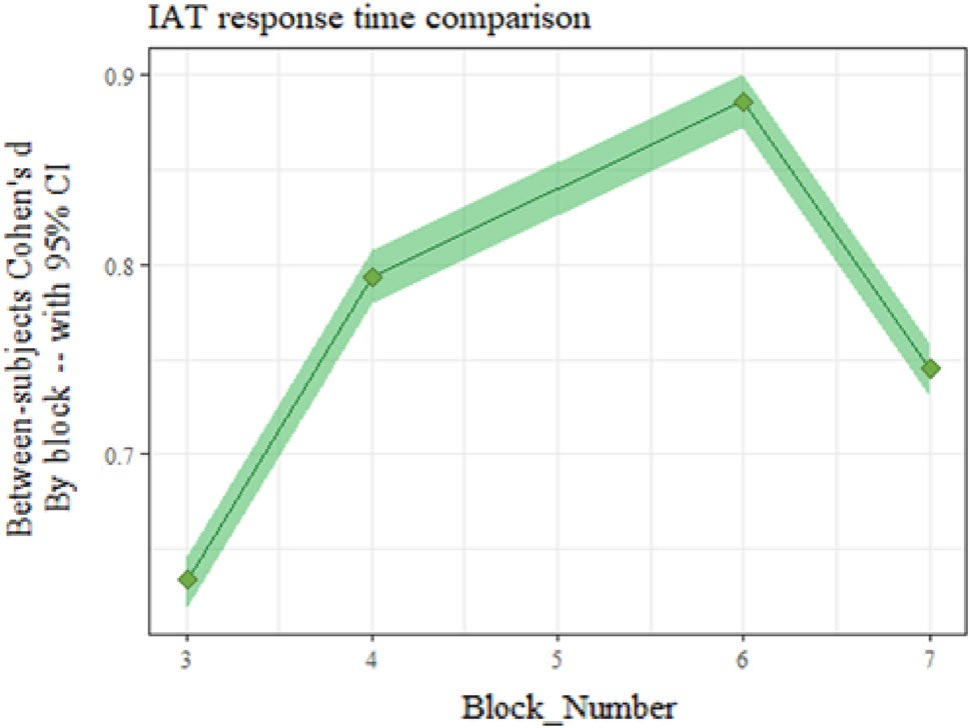
Between-subjects Cohen’s d of IAT response time differences. These are the standardized differences between distributions of response times, comparing between participants completing compatible vs incompatible blocks. In other words, the block 3 Cohen’s d compares the block 3 data for incompatible-first participants to the block 3 for compatible-first participants. The effect size of the between-subjects difference between compatible and incompatible stimuli changes as the task progresses, providing further evidence for time-dependent changes such as learning or self-regulation. Effect size increases from block 3 to 4 but decreases from block 6 to 7. Note that block 5 does not have dual-stimulus data and is not analyzed here. Shaded area indicates 95% CI of Cohen’s d.

The changing pattern was also seen in between-subjects contrasts of response times in the same block (i.e., comparing Block 4 RTs between participants whose Block 4 was a compatible block vs participants whose Block 4 was incompatible). The effects were large enough that error bars or t-values would convey little information. Instead, in Fig. 3, we show *d*_Cohen_ on each experimental block, providing a standardized estimate of the differences between trial types by block. The pattern of change is nonmonotonic, with blocks 4 and 6 having similar *d*_Cohen_ (i.e., between 0.74 and 0.80) while there was a smaller effect in block 3 (i.e., the first block of the first response pattern). In contrast, the most discriminative block was the first block of the second response pattern (i.e., highest effect size in block 6). These results, in conjunction with the order effects just presented, indicate a pattern in which the transition from a given combination (i.e., White + Good/Black + Bad to Black + Good/White + Bad) to a new combination leads to larger latencies. Even more notably, the first 20 trials showed much more variation in effect size than the last 40 trials (see Fig. 3, block 3 vs. 6 in comparison to block 4 vs. 7), implying that meaningful between-person variation in IAT scores may be captured by considering all (as recommended by Greenwald et al.^14^).

### Discussion

Each result reported under Study 1 indicates that IAT performance varies as a function of time within the task. More specifically, trials late in a session were systematically different from identical trials earlier in the same session. Response times decreased from early to later trials. Decreases in RT with experience may have been due to learning that was independent of implicit race bias, but the asymmetric order-dependent sizes of IAT D-scores demonstrated systematic experience-dependent changes in measured implicit evaluation. Asymmetric IAT effects due to order implicate time-dependent processes (e.g., learning, self-regulation, priming) that are not able to be addressed by conventional aggregated approaches to the IAT (i.e., D-score calculated from all trials). To overcome this limitation, in the following two studies we first demonstrate a method for fitting continuous IAT difference scores using by-trial generalized nonlinear mixed-effects regression. Next, we show that the relations between real-world measures of behavior and the IAT can be attributed at least partially to specific aspects of changing IAT difference values, thus providing a route to mechanistic specificity to observed effects.

## Study 2

The results of Study 1 indicated that there are temporal dynamics in IAT task performance. In Study 2 we address the feasibility of using by-trial time-sensitive analyses^12,22^ as a more accurate description of participant behavior than is achieved by a standard aggregation approach. To this end we used a sample of race IAT data, with every trial’s response time being available to enter into a nonlinear mixed-effects model of change over time. Model convergence (See Supplemental Information), clear patterns of change across trials, and reliable differences between trial types each indicated that such a modeling approach is viable to draw inferences regarding IAT performance.

### Method

#### Data source

Trial-wise data of racial bias IAT reported in Cox^16^ were used for this analysis. All participants in Studies 2 and 3 provided written informed consent and all procedures complied with a protocol approved by the University of Wisconsin-Madison Institutional Review Board. All methods were performed in accordance with approved protocols, relevant regulations, and the Declaration of Helsinki. Participants (N = 181) completed IATs online. In this and the following experiment, lower RT cutoffs were iteratively determined by testing the lowest RT on which participants performed above chance^32^; all RT below the resulting cutoff (320 ms; 1%) were excluded from analysis. Trials were likewise excluded for RT over 2000 ms (3%) or incorrect responses (6.7%; note that evidence-accumulation model extensions of the current methods would allow for a unified analysis of correct and incorrect responses^33,34^). Next, 6 participants were excluded for accuracy below 80% correct or for having at least 10 responses outside the included RT window; this left 175 participants with at least 90 trials each.

#### Analysis

Response times tend to be well fit by skewed distributions (for which assuming normality is problematic^35^), with the exponentially modified Gaussian distribution being particularly well-suited to analysis and interpretation of response times^36,37^. We thus modeled response times as an ex-Gaussian distribution with an exponentially-distributed decision process overlayed by a Gaussian mean and variance of additive noise^22^. As response times were allowed to parametrically vary (e.g., due to stimulus type or as a function of time), these parameters modified the exponential component. One benefit of this parameterization is the natural fit with the ubiquitous observation that response time variance is changed proportionally to the change in mean^38^.

Using ex-Gaussian nonlinear mixed-effects regression, RTs for compatible and incompatible trials were modeled as exponentially saturating functions of time^22,39,40^. By utilizing all combined-stimulus blocks of IAT trials, this provided two runs of 60 trials for each participant’s IAT data. The three parameters of the exponential learning function (starting level, rate of change, and asymptotic level) were each allowed to vary within each participant, by whether the block’s stimulus pairing was “Black + good/White + bad” or “White + good/Black + bad” (i.e., trial type; compatibility), and whether the block of trials was the second or the first. Nonlinear mixed-effects Bayesian model fitting utilized **rstan** via the **brms** package in R^41^. Start, rate, and asymptote parameters were each sampled on log scales (i.e., exponential link function), with rate being a time constant (i.e., number of trials to reach 50% of change in RT; see Supplemental Methods for model specification). Because rate was a time constant, smaller values of the rate parameter mean change happened in fewer trials, with larger values indicating that change took more time to occur.

Statistical reliability of parameters was determined by comparing the estimated parameter distributions’ CI to 0. In addition, a model was run with no change over time but otherwise identical parameterization (see Supplemental Information); this model was used to assess the overall value of the time-sensitive model compared to a model aggregating over time. Relative model fit was assessed the Leave-One-Out Information Criterion (LOOIC), an efficient approximation to leave-one-out cross-validated deviance^42^. Heuristic “reliable differences” are present when absolute differences are greater than 4 and are several standard errors of magnitude.

Models were parameterized (using start, rate, and asymptote) to have clear, yet tentative, interpretations regarding the processes giving rise to RT differences. Specifically, we considered that initial RT differences may index a participant’s “initial bias,” or their immediate tendency to respond differentially quickly to certain combinations of stimuli (e.g., slower to Black + good compared to White + good). Rate of change in RT differences may index a participant’s “self-regulation,” or their ability to change their own behavior with experience. Asymptotic RT differences may index a participant’s “persistent bias,” or the difference in response times to different stimulus types that remains even after the process of self-regulation. Thus, rather than extracting one index of IAT performance from a person’s data, three distinctly (if preliminarily) interpretable indices would be extracted. At statistical as well as conceptual levels, these three indices may be intercorrelated, and it is likely that at least one would be highly correlated with overall D score.

### Results

**Figure 4:**
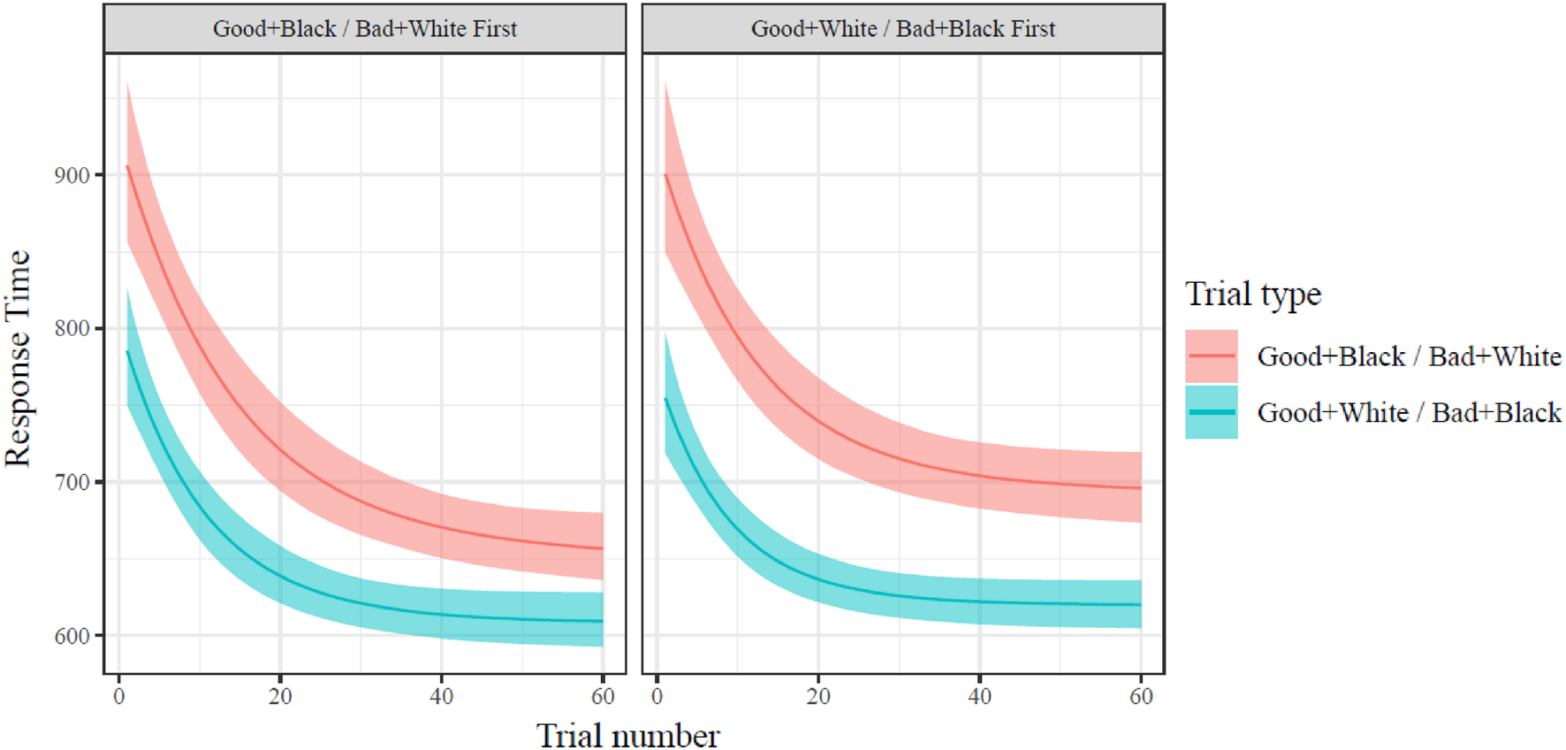
Model fit values of response times on compatible and incompatible trials in Study 2. Response times decrease over the course of each block, with a smaller decrease in incompatible trials when in the second block (i.e., participants in right panel have higher asymptotic RT than participants in left panel). Mean and 95% CI of fit values indicated.

All models showed adequate convergence indices (see Supplemental Information). The model of response time change over the experiment fit much better than the model without parameters of change over time (ΔLOOIC Mean = −685.2; SE = 63.5). There was a modest decrease in the distribution of response time differences (i.e., “Black + good & White + bad” vs. “White + good & Black + bad”) from early trials to late trials (d_Cohen_ = −0.57, CI = [− 0.60, − 0.54]; see also Table 1). This can be visually seen by comparing the difference between trial types on trial 1, in comparison to the difference in trial types on trial 60, in Fig. 4.

**Table 1:** Study 2 by-participant estimate means, CI, and correlations. All columns to the right of the 97.5% CI are product-moment correlations.

Parameter	Coefficient	Estimate	2.5% CI	97.5% CI	gaussianMean_Intercept	gaussianMean_WordVsFace	Start_Intercept	Start_Incongruent	Asymptote_Intercept	Asymptote_Incongruent	Rate_Intercept
log Gaussian Mean	Intercept	6.161	6.146	6.176							
	Word versus Face	0.127	0.118	0.136	− 0.337						
log Start	Intercept	5.878	5.856	5.902	0.294	− 0.053					
	Incongruence	0.374	0.343	0.405	− 0.117	− 0.116	− 0.205				
log Asymptote	Intercept	5.112	5.082	5.142	− 0.04	0.233	0.224	− 0.134			
	Incongruence	0.342	0.31	0.374	− 0.119	− 0.091	− 0.019	0.333	− 0.066		
log Rate	Intercept	2.451	2.288	2.606	0.149	0.194	0.523	− 0.111	0.606	0.098	
	Incongruence	0.658	0.618	0.699	− 0.123	− 0.122	− 0.061	0.688	− 0.024	0.685	− 0.011

**Figure 5:**
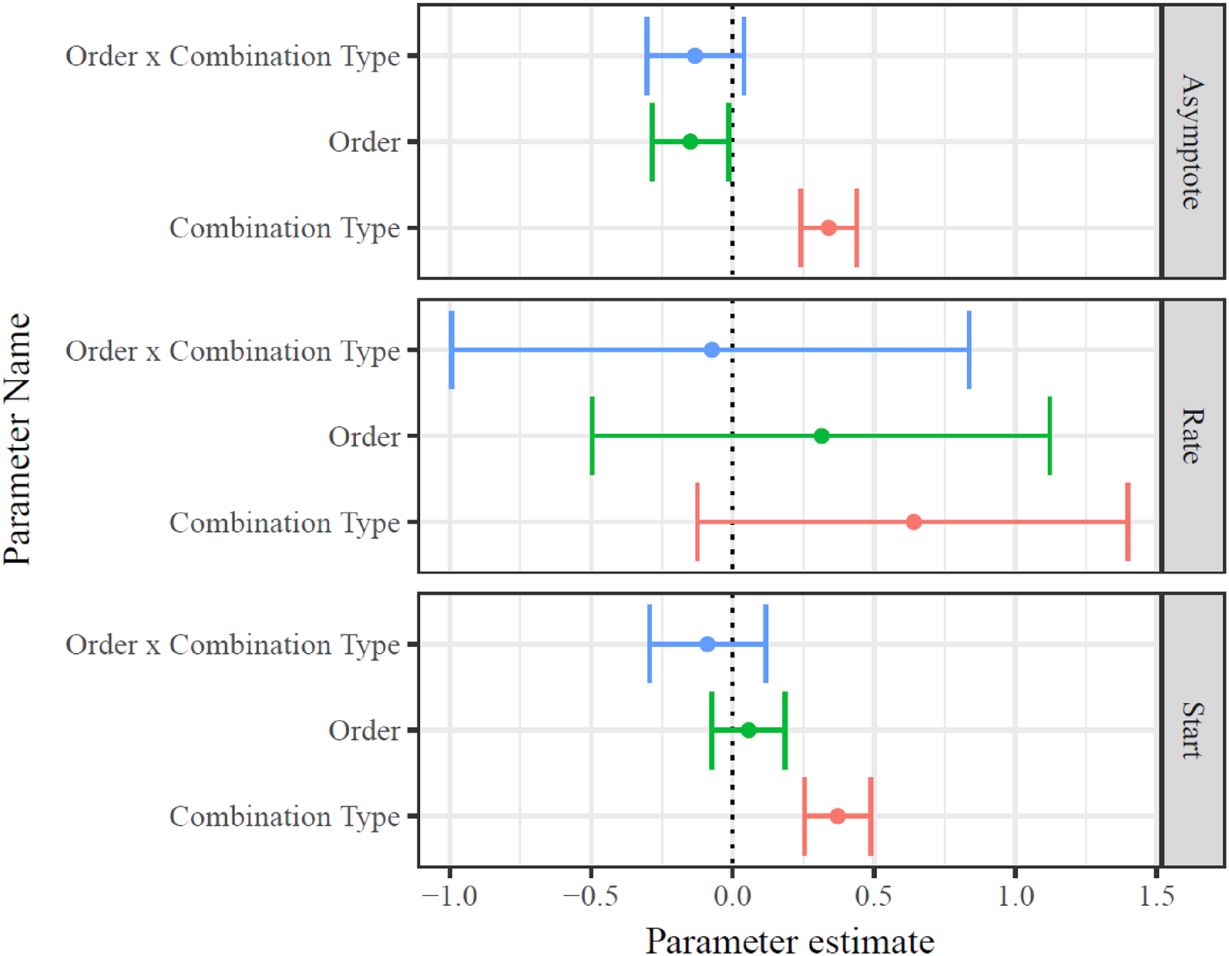
Distributions of model fit parameters of Study 2. Combination type indicates effect of incompatibility (i.e., “IAT effect”), in other words, the difference between compatible (White + Good and Black + Bad) blocks and incompatible (White + Bad and Black + Good) blocks. Order indicates whether a participant completed their incompatible or their compatible blocks first. Apart from main effects of incompatibility, only the main effect of Order on asymptotic RT was reliable. While the interactions between Order and Combination type in predicting Asymptotic RT and Starting RT replicated the direction of Study 2, the effects were not reliable in this sample of 175 participants.

Fixed effects indicated expected differences between compatible and incompatible trials both in starting response times (b = 0.374, CI_95_ = [0.343, 0.405] and asymptotic response times (b = 0.342, CI_95_ = [0.310, 0.374]; see Fig. 5, top and bottom panels). It should be reiterated that these values are log means of the exponential portion of the ex-Gaussian distribution, with the Gaussian portion remaining constant across all trials, and therefore the reported values represent the changes in both mean and variance in RTs commonly associated with compatibility differences and with learning. Notably, there were no differences in rate of change across trial type or presentation order (see Fig. 5, middle panel). In line with the aggregated analyses reported in Study 1, asymptotic response time was reliably lower when the Black + good/White + bad condition was first, but this condition order was not associated with reliable differences in starting (b = −0.089, CI_95_ = [− 0.294, 0.117]) or asymptotic (b = −0.133, CI_95_ = [− 0.303, 0.041]) compatibility effects.

### Discussion

Study 2 not only supported the results of Study 1 (i.e., that there are considerable temporal dynamics in IAT task performance), but also showed that it is possible to model these dynamics to create a more accurate description of performance. A host of interesting dynamics were observed, in particular that as response times decreased with task experience, the magnitude of the IAT score also decreased. More specifically, systematic changes in RT over 60 trials, including a disproportionate decrease in “Black + good & White + bad” RT led to a decrease in difference score with a moderate effect size. In addition, the model indicated a lack of a reliable effect on any change-related parameter of the interaction between trial type and order.

Indeed, variations between participants in aggregate IAT scores may arise from disparate sources, as indicated by Studies 1 and 2. Individual-level variation in IAT performance, as modeled in Study 2, could arise from variation in initial IAT score, rate of change in IAT score, or asymptotic IAT score. Each of the three components of changing IAT scores could be representative of different processes of learning within the task (e.g., rate of change may be related to self-regulation of prejudicial behaviors). In the following experiment, we explore the degree to which variations in time-related IAT score parameters may be related to other measures of bias such as behavioral discomfort when discussing sensitive topics. None of these specific tests would be possible using standard aggregated approaches to individual differences in IAT performance.

## Study 3

Study 2 provided evidence that it was appropriate to use a nonlinear ex-Gaussian mixed-effects model for characterizing trajectories of change in the IAT. However, such a characterization may be superfluous, if the parameters fit by such models do not provide any explanatory power beyond that of the typical overall IAT score. If patterns of associations between external measures and model parameters exist, they could provide two additional points of leverage. Parameters may specifically mirror overall IAT scores, thereby giving more specific information about the source of behavioral variation that is leading to associations with overall IAT scores (e.g., if IAT scores and asymptotic trial-type differences hold the same patterns, then it is likely that overall IAT scores are reflecting persistent bias). In contrast, if model parameters have specific associations with other measures that are not reflected in overall IAT scores, this would indicate that the aggregation of all trials in overall IAT scores may actually be obscuring information about participants’ performance that is independently indicative of important components of implicit evaluation. We seek out evidence for each of these latter two conclusions by comparing participant-level parameter estimates to those participants’ behaviors during an interview about race with an experimenter.

### Method

#### Data source, participant, and design

Data related to racial bias IAT was acquired from Cox^16; Study 3^. That study’s original purpose was to assess several interacting predictors of racial and gender biases (noting here we focus only on the racial component of the study). Specifically, the researcher was interested in how IAT bias interacted with two explicit individual difference measures, internal and external motivation to respond without prejudice, to predict behavior in interpersonal discussions. That is, this previously-reported work used interpersonal behavior as a criterion variable with which to assess interactions between IAT scores and motivations to respond without prejudice.

White participants (N = 188) discussed their opinions on race/racism and gender/sexism in the United States with an experimenter while being covertly videotaped. The race of the discussion partner was experimentally manipulated, with participants discussing race and gender issues with either a White person or a Black person, between-subjects.

#### Good/bad and competent/incompetent IATs

In addition to the standard IAT reported above, participants also completed a race IAT that paired Black and White faces with words related to Competence and Incompetence.

#### Internal and external motivations to respond without prejudice

In a mass survey prior to the study, all participants completed several questionnaires. This survey contained the internal motivation to respond without prejudice scale (IMS^18^). The IMS measures personal convictions in favor of nonprejudicial behavior (e.g., “I am personally motivated by my beliefs to be non-prejudiced toward Black people”). The scale has five items, scored on 9-point Likert scales (1 = *Strongly Disagree*, 9 = *Strongly Agree*).

External motivation to respond without prejudice scales (EMS) for prejudice against Black people^18^ was collected to measure sensitivity to social pressures against prejudice. The EMS items measure participants’ external (social, normative) motivations for behaving in a non-prejudiced way (e.g., “I try to hide any negative thoughts about Black people in order to avoid negative reactions from others”), and contains five items scored on 9-point Likert scales.

#### Procedure

Participants were randomly assigned to complete the study with one of three experimenters (a White male, Black male, or White female). The experimenter met the participant and led them into a faculty office where the experiment took place. Participants sat at a desk to read and sign a consent form and complete an initial “charged topics” questionnaire, which asked them to report their comfort discussing a number of different topics, including the topic they would later discuss, “Race relations in the United States”. Participants were led to believe that they would randomly select two of the topics to discuss, by drawing a topic out of each of two bowls filled with slips of paper with the topics written on them. In reality, the first bowl of topics contained only the race topic prompt, thus all participants discussed race. The race topic prompt read, “Discuss your opinions about race relations in the United States (for example: racial profiling, affirmative action or immigration laws in America, or whatever you feel is important).” After drawing their topics, participants were given a few minutes to jot down their thoughts on a “notes sheet,” for use during the discussion. They were told that they would share their opinions and perspectives on each topic for two minutes.

#### Seating distance

Once the participant was ready to begin, the experimenter casually asked them to bring the chair over to start the discussion. The participant’s placement of the chair relative to the experimenter provided a measure of seating distance, a common indicator (i.e., criterion variable) of interpersonal comfort/closeness that has previously correlated with IAT^43^. After the discussion concluded and the participant left, the experimenter measured the distance between the chairs at three reference points: one for the centers of the chairs, and one for each side of the chairs. These three measurements, measured in centimeters, were averaged into a single seating distance score (Cronbach’s α = 0.922).

#### Discussion and experimenter ratings

Participants discussed the race topic for a full two minutes. After the discussions, the experimenters rated participants on how racist they seemed using 1-to-5 Likert scales. The single ratings of how racist/nonracist the participant seemed to the experimenter served as the dependent variable “Seeming Racist.”

#### Analysis

From the initial 188 participants we proceeded to exclude people for several reasons. Thirty-six participants were excluded for missing variables of interest (i.e., seeming racist, seating distance, EMS, or IMS). An additional 7 participants were excluded for being multivariate outliers among the race variables of interest. This was determined using a robust Mahalanobis distance method^44^. Robust covariance estimation used a minimum 80% of cases, and outlier rejection used an alpha of 0.01. Participant rejection left 146 participants remaining, which would be associated with an 80% power to detect a product-moment correlation of at least 0.23. No participants were rejected solely due to IAT performance; minimum number of included trials per participant was 190 (i.e., 79.2% of trials retained after exclusion due to RT or accuracy criteria).

Time-sensitive indices of IAT performance were estimated using nonlinear generalized mixed-effects models of by-trial change in RT, which were parameterized and fit similarly to Study 2 (see Supplemental Information for model specification). Trial-wise exclusions used the same lower RT threshold as empirically determined in Study 2 (lowest remaining RT 324 ms). However, two key changes were made. First, both IATs were fit in a single model (see also Study 4 for a similar procedure). IAT type (Good/Bad or Competence/Incompetence) was included as a zero-centered dichotomous predictor in both fixed and random effects, providing for estimation of time-dependent components of RT difference on either IAT type or at an intermediate level. Second, the effect of order was removed from the model due to the lack of reliable effects of order on RT differences in Study 2, as well as to reduce model complexity.

As in Study 2, Study 3 utilized a 3-parameter model of by-trial change in RT. Participants’ RT, including the differences between trial types, were modeled as arising from an ex-Gaussian distribution with a Gaussian noise component and an exponential decision component which itself had starting values, rates of change over the course of the experiment, and asymptotic values. The participant-level point estimates extracted from the model random effects for each of these parameters were used as indices of IAT performance, with potentially distinct interpretations, in analyses of Study 3 (see Supplemental Fig. S1 for correlations between IAT indices). Like in Study 2, a comparison null model was also fit with no change over time.

Following previously-reported results^16^, links between indices of IAT performance were tested in relation to real-world behaviors such as seeming prejudiced or seating distance from an experimenter while discussing a sensitive topic. We used bootstrapped robust linear regression to test the reliability of main effects of IAT variables, as well as interactions with EMS and IMS scores and experimenter sex or race. Regression models were fit using the *tef_rlm_boot* function in the R package **TEfits**^45^ and 5,000 resamples with replacement to estimate coefficient 95% confidence intervals. Each index of IAT was standardized (z-scored) prior to bootstrapped linear model fitting in order to maintain the same scale for coefficients across indices.

### Results

#### Trial-based IAT

**Figure 6:**
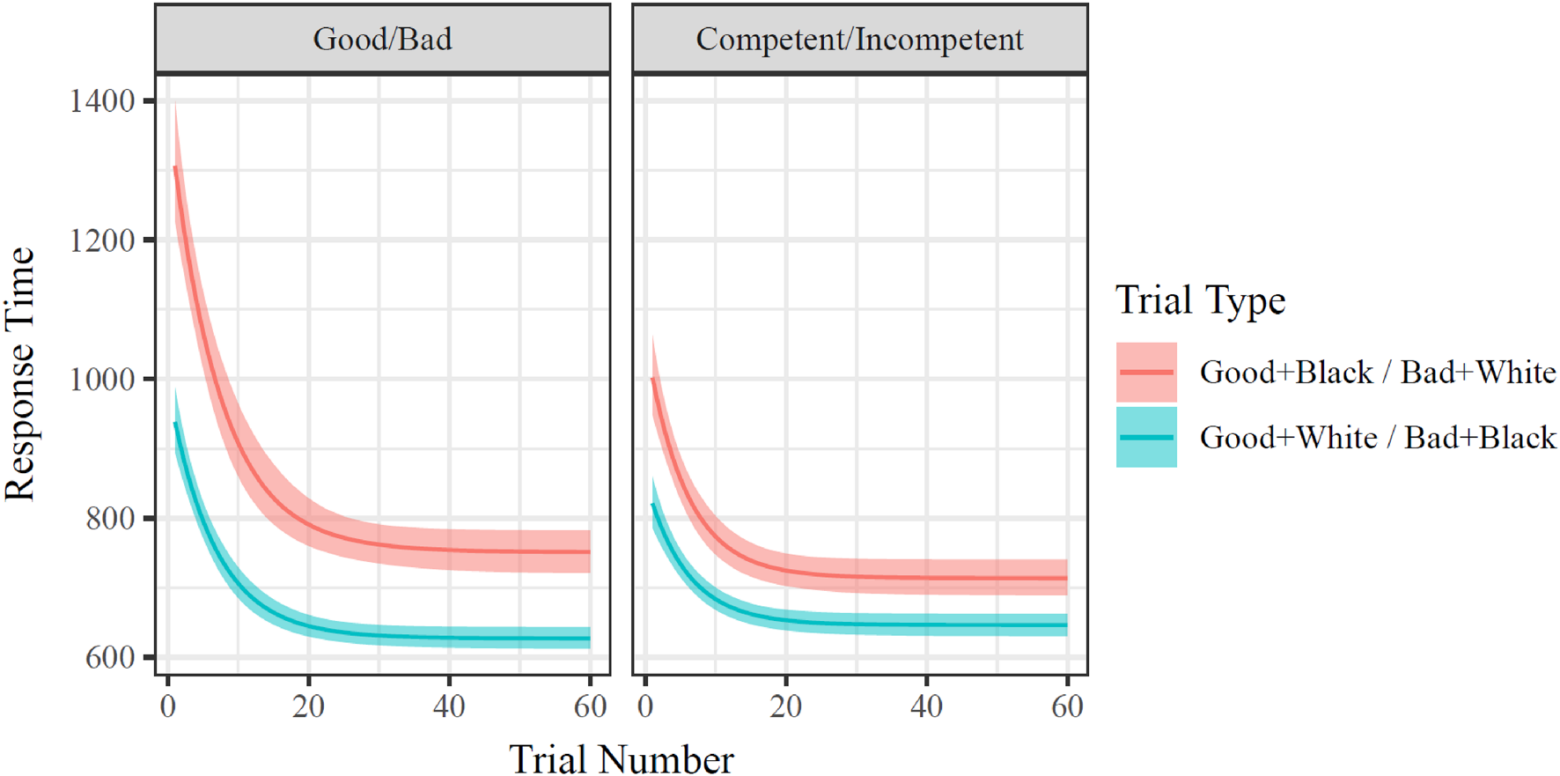
Group-level by-trial model fits of Study 3. Response times decreased in both IAT types and for both trial types, with group-level changes appearing to plateau by around trial 30. Solid lines indicate means and shaded areas indicate 95% area of predicted RT.

The model including changes over time fit much better than the model which fit response times as constant over the course of the experiment (ΔLOOIC Mean = −2210.5, SE = 125.6). Race IAT trial type difference scores were reliable (with reliability assessed indicated by 0 falling outside the 95% CI of estimated Start and/or Asymptote parameter fixed effects for trial type). Overall participant-level Race IAT trial type effects are used in subsequent analyses because the effect of trial type was evident, and the same numerical direction, in both Race IAT types (see also Study 4 for the use of multiple IATs in estimating effects). Further, using both tasks to estimate individuals’ parameters should increase those parameters’ precision (which was the reason to estimate both IAT types in the same model). Still, it is notable that the Good/Bad Race IAT showed larger trial type effects than the Competent/Incompetent Race IAT (see Fig. 6).

RT differences tended to decrease when assessed using participant-level parameters (i.e., reduction in trial type difference over time; d_Cohen_ = −0.37; see Table 2 for descriptive statistics of participant-level estimates). Wilcoxon sign-rank tests indicated significantly lower participant-level asymptotic than starting RT differences (med_asym-start_ = −0.05, W = 3250, Z = −3.65, *p* < 0.001). These results recapitulated the qualitative pattern of both Study 1 and Study 2, namely, reductions in RT over time alongside reductions in IAT effect magnitudes.

**Table 2:** Study 3 by-participant estimate means, CI, and correlations. All columns to the right of the 97.5% CI are product-moment correlations.

Parameter	Coefficient	Estimate	2.5% CI	97.5% CI	gaussianMean_Intercept	gaussianMean_WordVsFace	Start_Intercept	Start_IATtype	Start_Incongruence	Start_IATtype:Incongruence	Asym_Intercept	Asym_IATtype	Asym_Incongruence	Asym_IATtype.Incongruence	Rate_Intercept	Rate_IATtype	Rate_Incongruence
log Gaussian Mean	Intercept	6.128	6.114	6.141													
	Word versus Face	0.153	0.146	0.162	− 0.291												
log Start	Intercept	6.035	5.983	6.086	0.123	0.165											
	IAT type	− 0.28	− 0.287	− 0.274	0.037	− 0.094	− 0.045										
	Incongruence	0.487	0.483	0.491	− 0.082	− 0.06	0.026	0.045									
	IAT type : Incongruence	− 0.165	− 0.173	− 0.156	0.026	− 0.031	0.119	0.598	0.005								
log Asymptote	Intercept	5.184	5.133	5.232	0.037	0.23	0.571	0.087	− 0.016	0.134							
	IAT type	0.106	0.074	0.138	− 0.182	0.14	− 0.047	0.169	− 0.023	− 0.059	0.038						
	Incongruence	0.428	0.399	0.458	0.031	0.079	0.001	0.037	0.257	0.082	− 0.212	− 0.024					
	IAT type : Incongruence	− 0.243	− 0.272	− 0.214	− 0.057	0.056	0.042	− 0.05	0.051	0.101	0.083	− 0.187	− 0.038				
log Rate	Intercept	0.928	0.744	1.114	0.155	0.201	0.403	0.005	− 0.149	0.159	0.236	0.005	− 0.07	0.163			
	IAT type	− 0.351	− 0.42	− 0.28	− 0.116	0.094	− 0.053	0.594	0.044	0.196	0.089	0.32	0.036	− 0.045	− 0.105		
	Incongruence	0.071	− 0.091	0.231	− 0.065	0.035	− 0.201	0.05	0.566	− 0.156	− 0.27	0.191	0.4	− 0.18	− 0.133	0.147	
	IAT type : Incongruence	− 0.279	− 0.294	− 0.264	0.007	0.04	0.185	0.222	− 0.02	0.624	0.152	− 0.151	0.03	0.441	0.237	0.224	− 0.289

To assess our by-participant estimates, analyses testing the findings described in the original reporting of this data^16^; Study 3 were separately conducted using 4 IAT-derived measures (i.e., overall score and 3 time-dependent parameters). That is, the IAT scores entered into the regressions we report were either overall measures or single parameters describing aspects of the time-evolving IAT score. All plots below organize different models’ parameters into figure panels, with the measure of IAT (and likewise the specific model) color-coded. Plots show means and bootstrapped 95% CI of robust regression coefficients, with coefficient reliability being indicated by 0 being outside of 95% CI (see also Tables 3, 4, 5). Only coefficients including an index of IAT performance were included in plots.

**Table 3:** Boostrapped robust linear models’ coefficients for Study 3, predicting Seeming Racist.

IAT_measure	Predictor	Value	2.5% CI	97.5% CI
Overall score	1. IntervWhite	− 0.038	− 0.391	0.309
	2. IAT	− 0.154	− 0.346	0.1
	3. IMS	0.127	− 0.105	0.325
	4. IntervWhite:IAT	0.117	− 0.261	0.424
	5. IntervWhite:IMS	0.039	− 0.271	0.375
	6. IAT:IMS	0.086	− 0.133	0.328
	7. IntervWhite:IAT:IMS	− 0.088	− 0.428	0.221
Starting score	1. IntervWhite	− 0.016	− 0.379	0.315
	2. IAT	− 0.142	− 0.384	0.136
	3. IMS	0.155	− 0.124	0.394
	4. IntervWhite:IAT	0.037	− 0.347	0.391
	5. IntervWhite:IMS	0.02	− 0.314	0.403
	6. IAT:IMS	0.009	− 0.222	0.253
	7. IntervWhite:IAT:IMS	− 0.093	− 0.465	0.221
Change rate	1. IntervWhite	− 0.053	− 0.414	0.329
	2. IAT	− 0.184	− 0.371	− 0.001
	3. IMS	0.199	− 0.063	0.448
	4. IntervWhite:IAT	0.185	− 0.177	0.513
	5. IntervWhite:IMS	− 0.039	− 0.394	0.339
	6. IAT:IMS	− 0.012	− 0.174	0.184
	7. IntervWhite:IAT:IMS	0.055	− 0.277	0.319
Asymptotic score	1. IntervWhite	− 0.081	− 0.426	0.274
	2. IAT	− 0.01	− 0.241	0.217
	3. IMS	0.097	− 0.109	0.319
	4. IntervWhite:IAT	0.028	− 0.346	0.356
	5. IntervWhite:IMS	0.06	− 0.284	0.364
	6. IAT:IMS	0.249	0.041	0.444
	7. IntervWhite:IAT:IMS	− 0.141	− 0.52	0.149

**Table 4:** Boostrapped robust linear models’ coefficients for Study 3, predicting seating distance, among all participants.

IAT_measure	Predictor	Value	2.5% CI	97.5% CI
Overall score	1. IntervWhite	− 0.131	− 0.534	0.214
	2. IAT	0.152	− 0.159	0.441
	3. EMS	0.056	− 0.185	0.366
	4. IntervWhite:IAT	− 0.176	− 0.532	0.216
	5. IntervWhite:EMS	− 0.196	− 0.57	0.093
	6. IAT:EMS	− 0.271	− 0.58	0.009
	7. IntervWhite:IAT:EMS	0.598	0.231	0.957
Starting score	1. IntervWhite	− 0.094	− 0.525	0.287
	2. IAT	0.089	− 0.248	0.295
	3. EMS	0.048	− 0.172	0.379
	4. IntervWhite:IAT	− 0.101	− 0.411	0.282
	5. IntervWhite:EMS	− 0.158	− 0.544	0.16
	6. IAT:EMS	− 0.176	− 0.482	0.115
	7. IntervWhite:IAT:EMS	0.318	− 0.032	0.685
Change rate	1. IntervWhite	− 0.078	− 0.497	0.314
	2. IAT	0.09	− 0.208	0.49
	3. EMS	0.168	− 0.167	0.506
	4. IntervWhite:IAT	− 0.086	− 0.506	0.284
	5. IntervWhite:EMS	− 0.285	− 0.667	0.107
	6. IAT:EMS	− 0.313	− 0.66	− 0.009
	7. IntervWhite:IAT:EMS	0.568	0.19	0.973
Asymptotic score	1. IntervWhite	− 0.11	− 0.474	0.242
	2. IAT	0.109	− 0.228	0.411
	3. EMS	0.038	− 0.219	0.36
	4. IntervWhite:IAT	− 0.149	− 0.507	0.284
	5. IntervWhite:EMS	− 0.159	− 0.515	0.172
	6. IAT:EMS	− 0.166	− 0.463	0.1
	7. IntervWhite:IAT:EMS	0.423	0.084	0.796

**Table 5:** Boostrapped robust linear models’ coefficients for Study 3, predicting seating distance, among participants with a Black experimenter.

IAT measure	Predictor	Value	2.5% CI	97.5% CI
Overall score	1. IAT	0.146	− 0.158	0.424
	2. EMS	0.088	− 0.18	0.393
	3. IAT:EMS	− 0.275	− 0.595	0.006
Starting score	1. IAT	0.072	− 0.305	0.316
	2. EMS	0.066	− 0.185	0.374
	3. IAT:EMS	− 0.178	− 0.474	0.08
Change rate	1. IAT	0.089	− 0.216	0.422
	2. EMS	0.186	− 0.118	0.525
	3. IAT:EMS	− 0.326	− 0.666	− 0.027
Asymptotic score	1. IAT	0.1	− 0.221	0.402
	2. EMS	0.064	− 0.213	0.397
	3. IAT:EMS	− 0.157	− 0.445	0.092

Thus, here we turned to comparing the conventional reporting of the data, originally in Cox^16^, to the use of by-participant estimates of components of RT change. Cox^16^ showed a 3-way interaction between IAT, IMS, and experimenter race when predicting “seeming racist” – driven by the white-experimenter condition (B = −0.608, β = −0.288 , t = −1.994, p = 0.048). There was also a 3-way interaction between IAT, EMS, and experimenter race when predicting seating distance – driven by the black-experimenter condition (B = 5.910, β = 0.476 , t = 3.211, p = 0.002 ). Finally, there was no bivariate relationship between IAT and seat-distance-to-black-person.

**Figure 7:**
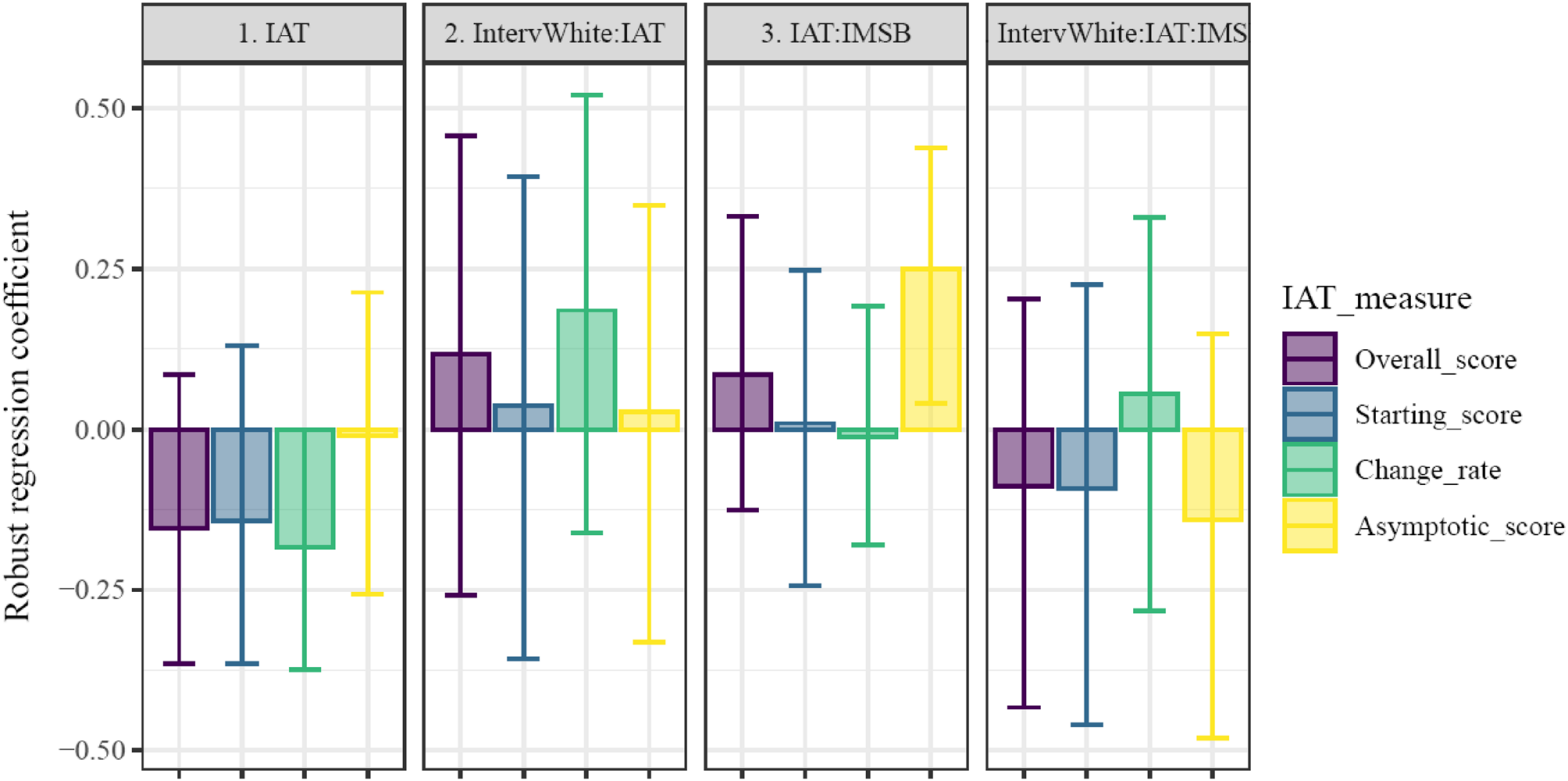
Robust regression coefficients predicting participants’ Seeming Racist. Results of 4 robust linear models, each using a different z-scored index of IAT performance. A reliable interaction was evident with internal motivation to respond without prejudice. This two-way interaction was only reliable when using asymptotic RT difference as the measure of IAT. All other measures of IAT RT difference did not show reliable prediction of Seeming Racist. Models are denoted by color, with each panel showing the analogous coefficients across models. Bar heights indicate coefficient means, with error bars representing bootstrapped 95% CI.

Examining these patterns using time-sensitive indices, reliable effects were observed in predicting z-scored Seeming Racist using different indices of IAT performance (see Fig. 7; Table 3). No coefficients were reliable in the model involving starting RT differences. In the model using rate of change in IAT to predict Seeming Racist, only the main effect of rate of change was reliable (b = −0.18, CI_boot_ = [− 0.37, − 0.01]), indicating that faster changes in RT differences were associated with more Seeming Racist. In contrast, in the model using asymptotic RT differences as the index of IAT performance, a reliable interaction with IMS was present (b = 0.25, CI_boot_ = [0.04,0.44]), indicating that participants with larger RT differences at the end of the task had a stronger link between Seeming Racist and IMS.

**Figure 8:**
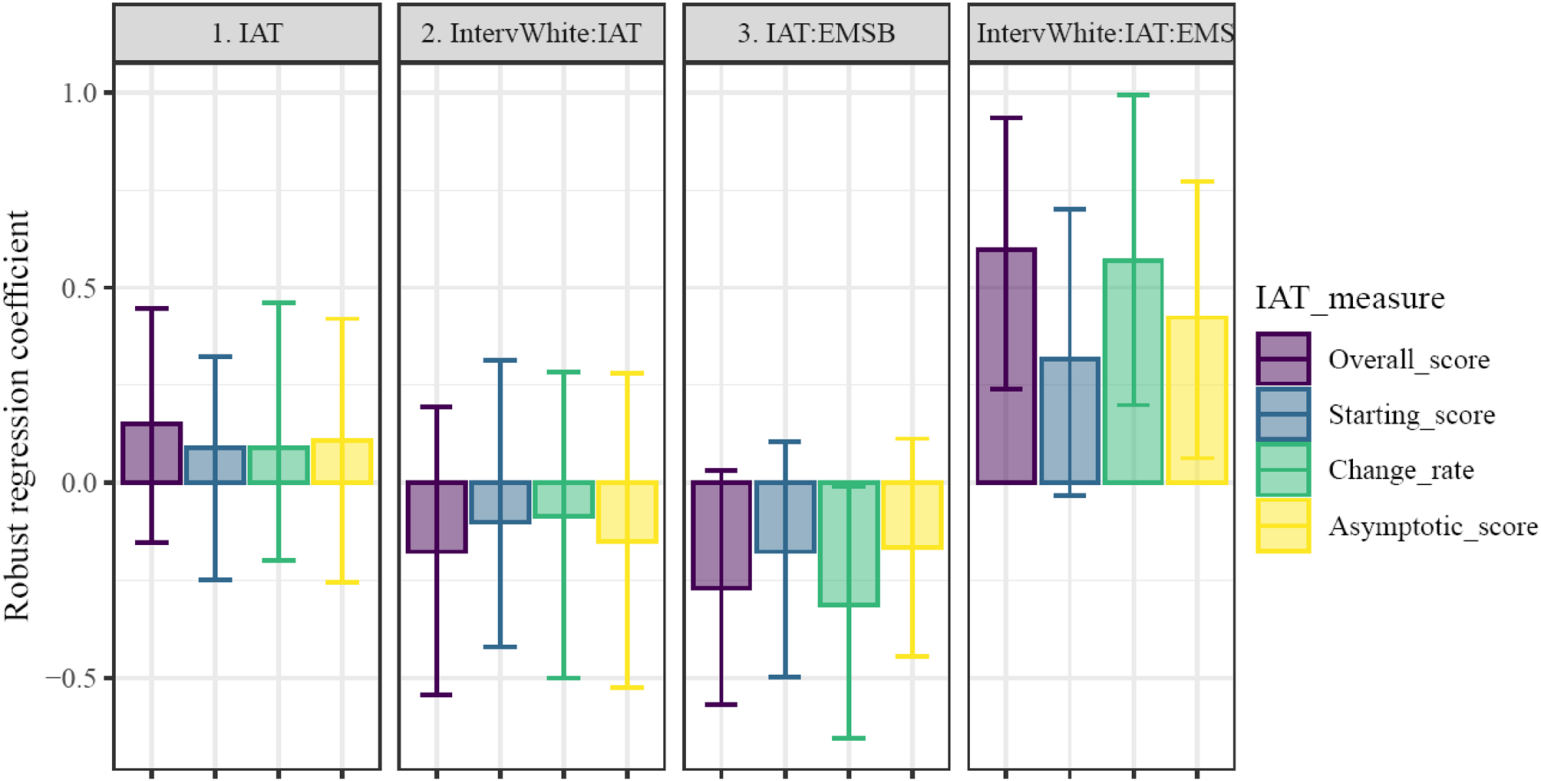
Robust regression coefficients of IAT measures’ predictiveness of seating distance within all participants. Results of 4 models, each using a different index of IAT performance. Reliable interactions were evident with interviewer race and external motivation to respond without prejudice. This three-way interaction was reliable when using overall RT difference, using the rate of change in RT difference, or when using asymptotic RT difference as the measure of IAT. Models are denoted by color, with each panel showing the analogous coefficients across models. Bar heights indicate coefficient means, with error bars representing bootstrapped 95% CI.

We next examined relations between IAT measures and z-scored Seating Distance from the experimenter. Several reliable effects were observed in predicting Seating Distance from the experimenter (see Fig. 8; Table 4). The results of predicting Seating Distance were replicated^16^, with a reliable interaction between interviewer race, overall IAT score, and EMS (b = 0.60, CI_boot_ = [0.23,0.96]). In a parallel to the effects of overall IAT score, the rate of change in RT differences was a reliable predictor of Seating Distance in the three-way interaction with interviewer race and EMS (b = 0.57, CI_boot_ = [0.20,0.97]) as well as the two-way interaction with EMS (b = −0.31, CI_boot_ = [− 0.66, − 0.01]). These results indicate that faster changes in RT were associated with stronger links between EMS and Seeming Racist, with this pattern being moderated by the race of the interviewer. The three-way interaction between asymptotic RT difference, EMS, and interviewer race was likewise reliable (b = 0.42, CI_boot_ = [0.08, 0.79]). No reliable effects were evident in models using starting RT differences as indices of IAT performance.

To investigate further the effects of IAT in predicting Seating Distance from the experimenter, Cox^16^ tested a model including only participants with a Black experimenter. Unlike the previously-reported results, in our analyses the interaction between overall IAT score and EMS was not reliably predictive of Seating Distance (b = −0.28, CI_boot_ = [− 0.59,0.01]; see Table 5). When using rate of change in RT difference as an index of IAT performance the interaction between rate of change and EMS.B had a slightly larger magnitude than the interaction including the overall score (b = −0.33, CI_boot_ = [− 0.67, − 0.03]). These results confirmed that faster changes in RT differences were associated with stronger relations between EMS and Seating distance.

### Discussion

While overall IAT scores were reliably associated with behavioral measures in several models, particularly when interacting with measures of motivations to respond without prejudice, it was possible that these relationships were attributable to dissociable time-varying components of behavior in the IAT task. When using a time-dependent decomposition of RT differences, results showed that certain aspects of behavioral trajectories may be particularly related to real-world behaviors. In particular, RT difference rates of change as well as asymptotic differences were reliable predictors of behavioral measures across several models. These results indicate the potential utility of using time-evolving measures to clarify the relations between IAT scores and real-world behaviors. The lack of reliable associations with starting RT difference could be interpreted as support for considering early trials to be merely “practice”. This conclusion would be premature, however, because the early trials’ dynamics (i.e., the rate of RT differences’ change over time) were informative with regards to external measures.

Collinearity is a major limitation to the results we report here. We do not intend to imply that the various components of change in RT differences were independent, and in fact, they were correlated with one another (see Supplemental Information). In our results, then, components of change were partially redundant with overall scores. The differences in relations to non-IAT measures indicate that the different components were indeed capturing behavioral signatures of distinct aspects of IAT performance, or at very least they were differentially successful (e.g., through noise reduction) at capturing the same aspects underlying IAT performance that are linked to external measures. Further research that specifically targets the dynamics of IAT would be needed to identify uniquely predictive aspects of components of change.

Although Study 3 is concerned with individual differences in implicit evaluation, IAT and related methods are often also used to test between-group effects as well as we will see in Study 4 below.

## Study 4

Although we have considered whether the IAT itself shows within-session dynamics, as well as whether these dynamics have consequences for inferences regarding individual differences measures, there is another important facet to IAT research: namely, experimental manipulations meant to induce between-group differences in scores.

### Methods

Kurdi and Banaji^46^ presented a series of studies testing possible learning-related bases of response compatibility effects such as those assessed using the IAT. Briefly, they contrasted compatibility effects that had been induced using repeated evaluative pairings (i.e., associative learning of stimulus pairings; REP) versus evaluative statements (i.e., explicit learning of stimulus pairings; ES) with additional between-subject conditions for stimulus combinations which hadn’t been reinforced and those in which both ES and REP learning had occurred. Specifically, in their Study 3, which we reanalyze here, they focused on whether, within a single IAT session, compatibility effects learned through REP would decay more slowly than compatibility effects associated through ES. A sliding window was used to calculate conventional IAT D scores for small blocks of trials, and it was found that (a) ES and Combined conditions were reliably different than the control condition, (b) all conditions’ compatibility effects decreased over time, and (c) the magnitudes of ES compatibility effects decreased more from the beginning of the 40 trial block to the end of the 40 trial block.

Critically, while a sliding window of IAT D scores can allow researchers to roughly identify patterns of change over time, it is limited in several key ways. First, like all aggregation-based methods (e.g., calculating means, etc.) the windowed procedure implements the assumption that, within a given window, all trials are generated by the same underlying process (e.g., the compatibility effect is the same on trial 1 and trial 10 of each given window). While the authors mitigated this concern by also calculating windowed D scores for blocks of 5 trials a time and showing a qualitative similarity, we approach the changes by considering that the processes of interest are undergoing a trial-to-trial and saturating change. Further, by implementing a unified model to characterize compatibility effects and changes in the same estimation procedure, we are able to avoid intermediate steps such as iteratively using windows of trials and then fitting a model to test patterns in those windows’ results. Second, by enabling the use of each trial’s data, our approach allows both comparisons of compatibility effects (as would be considered using D scores) as well as an examination of the patterns of behavior giving rise to those compatibility effects (e.g., incompatible trials’ RTs changing more slowly than compatible trials’ RTs).

Here we recapitulate the reported results by fitting a response compatibility model, as with the IAT model in Studies 2 and 3 above, to experiments 3a through 3e of Kurdi and Banaji^46^. Specifically, we estimated trajectories of response time change as the exponential component of an ex-Gaussian distribution, with a Gaussian mean and variance that remained constant. The three parameters defining the trajectories of change (i.e., starting value, time constant of change, and asymptotic value) were each defined as the predicted values of independent and simultaneously-estimated generalized linear mixed-effects models with fixed effects of learning condition, stimulus “incompatibility,” and the interaction between condition and incompatibility, with zero-centered gender and zero-centered age as covariates (see Supplemental Information for model formula). Random effects were included for participant-level intercepts and compatibility slopes (i.e., compatibility effects). By-experiment random intercepts, and random slopes corresponding to each of the fixed effects, were also estimated. Using this framework, we were able to identify the differences in learning condition’s compatibility effects as they dynamically changed throughout the experiment (note that condition effects used the no-learning condition as the reference level, so all coefficients are interpretable as differences from this baseline).

### Results

**Figure 9:**
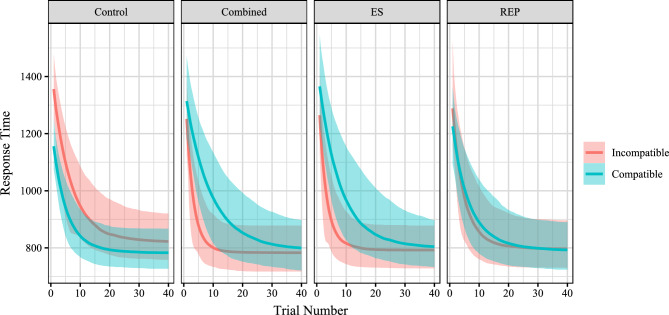
Fit response time trajectories of data from Kurdi and Banaji^46^. All conditions showed decreases in response times over the course of blocks. Note that compatible and incompatible refer to the baseline tendencies of participants, as indicated in the control condition, and are opposite to the response tendencies that were taught to participants in each of the other conditions.

The model of compatibility effect change converged, with all R-hats below 1.05, all fixed-effect R-hats below 1.03, and all fixed-effect Effective Sample Sizes above 95. Fixed-effect coefficients qualitatively followed the findings reported in Kurdi & Banaji (2019; see Table 6; Fig. 9). Specifically, in the control condition, a reliable incompatibility effect was observed in the beginning (b = 0.296, CI_95_ = [0.036, 0.555]) which decreased by the end (b = 0.163, CI_95_ = [− 0.317, 0.703]). The REP condition did not show a reliable difference from the control condition in compatibility effect in either the beginning (b = −0.195, CI_95_ = [− 0.449, 0.114]) or in the end (b = −0.139, CI_95_ = [− 0.412, 0.107]). In contrast, both other learning conditions showed reliable differences from the control condition (ES beginning: b = −0.431, CI_95_ = [− 0.660, − 0.206], ES end b = −0.163, CI_95_ = [− 0.357, − 0.019], Combined beginning b = −0.386, CI_95_ = [− 0.547, 0.206], Combined end b = −0.190, CI_95_ = [− 0.357, − 0.040]).

**Table 6:** Fixed effects of Study 4.

Parameter	Coefficient	Estimate	2.5% CI	97.5% CI	Reliability
log Start	Intercept	6.509	6.438	6.583	*
	Condition_Combined	0.049	− 0.056	0.17	
	Condition_ES	0.093	− 0.03	0.207	
	Condition_REP	0.011	− 0.108	0.143	
	Incompatibility	0.296	0.036	0.555	*
	Gender	− 0.016	− 0.167	0.123	
	Age	0.12	0.071	0.169	*
	Condition_Combined:Incompatibility	− 0.386	− 0.547	− 0.206	*
	Condition_ES:Incompatibility	− 0.431	− 0.66	− 0.221	*
	Condition_REP:Incompatibility	− 0.195	− 0.449	0.114	
log Rate	Intercept	1.91	1.091	2.535	*
	Condition_Combined	− 0.188	− 0.746	0.391	
	Condition_ES	− 0.254	− 0.912	0.408	
	Condition_REP	− 0.099	− 0.756	0.555	
	Incompatibility	0.341	− 1.67	1.876	
	Gender	0.363	− 0.165	0.818	
	Age	0.422	0.195	0.661	*
	Condition_Combined:Incompatibility	− 2.126	− 3.125	− 0.565	*
	Condition_ES:Incompatibility	− 1.961	− 2.879	− 0.601	*
	Condition_REP:Incompatibility	− 0.9	− 2.15	0.537	
log Asymptote	Intercept	5.431	5.231	5.646	*
	Condition_Combined	− 0.08	− 0.287	0.107	
	Condition_ES	− 0.038	− 0.177	0.078	
	Condition_REP	− 0.041	− 0.172	0.086	
	Incompatibility	0.163	− 0.317	0.703	
	Gender	0.029	− 0.078	0.126	
	Age	0.007	− 0.035	0.052	
	Condition_Combined:Incompatibility	− 0.19	− 0.357	− 0.04	*
	Condition_ES:Incompatibility	− 0.163	− 0.326	− 0.019	*
	Condition_REP:Incompatibility	− 0.139	− 0.412	0.107	
log Gaussian Mean	Intercept	574.046	552.896	593.185	*

Indicating the differential dynamics of change between compatible and non-compatible stimulus combinations, both ES (b = −1.961, CI_95_ = [− 2.879, − 0.601]) and Combined (b = −2.126, CI_95_ = [− 3.125, − 0.565]) conditions showed compatibility differences in the rate of change in RT. Interestingly, this pattern appears to be due to participants in the ES and Combined groups rapidly quickening in their incompatible trials (i.e., the trials taught to them to be compatible), whereas both control and REP groups appear to have trial types that change at approximately the same rates (see figure). Indeed, when directly comparing the coefficients’ posterior distributions between groups, incompatibility modulated rate of RT change more in ES than REP (b = 1.057, CI_95_ = [− 0.762, 2.787]), although this difference was not reliable. Similarly, ES showed a reliable decay in compatibility (i.e., difference between asymptote and start; b = 0.273, CI_95_ = [0.023, 0.526]) while REP did not reliably decay (b = 0.059, CI_95_ = [− 0.360,427]), and the difference between these two effects was not itself reliable (b = 0.214, CI_95_ = [− 0.220, 0.694]).

### Discussion

The results of Study 4 recapitulated the findings of Kurdi and Banaji^46^, showing that explicit learning provided larger learning of compatibility effects than did associative learning. Further, while ES-learned compatibility effects reliably decreased over the course of 40 trials, REP-learned compatibility did not decay over that time. This latter effect, as in the original article, took the form of a stable lack of difference between stimulus combinations (i.e., REP learning appeared to robustly unlink the associations apparent in the control condition). By constructing a model to fit trial-by-trial dynamics in all participants, conditions, and experiments simultaneously, we were able to test the core hypothesis presented in the previous paper in a unified model. Such a unified approach may be applied in other intervention approaches as well, with between-group contrasts having maximal power by considering data at a by-trial level while also partialling out nuisance sources of variance. As with Studies 2 and 3, such models can also be extended to test alternative models of stability or functional forms of change over time^39,47^.

## General Discussion

Across 3 studies, IAT effects demonstrated robust within-person changes during the task. Learning, in the form of reduced response times, was evident in an aggregated large dataset (Study 1) as well as using by-trial modeling methods (Studies 2 & 3). The uniformity of these time-dependent effects should prompt an investigation of whether these aspects of IAT, such as initially large incompatibility effects or rapid suppression thereof, could provide new insights to theories of implicit evaluation. As a preliminary investigation of this question, Study 3 examined individual differences in various “real-world” behaviors as associated with various indices of IAT performance, such as overall scores or components of time-dependent trajectories of change in RT.

When relating components of participants’ RT change with behavioral measures, there was recurring evidence for the importance of rate of change. This implicates possible individual differences in the efficacy of self-regulation processes, as opposed to initial “gut reactions” or asymptotic “persistent bias,” although all propositions regarding mechanisms are preliminary. For instance, in replicating the analyses of Cox^16^; Experiment 3, rate of change was qualitatively similar to overall IAT effect in its interaction with External Motivation to respond without prejudice (EMS) to predict seating distance from an experimenter, particularly when the experimenter was Black. Prior interactions between IAT and IMS/EMS, in predicting real-world behaviors, have been interpreted as evidence for self-regulation processes that may either allow or prevent implicit evaluation from becoming explicit evaluation. The results we reported here support this interpretation due to the uniformity of reliable effects of a regulation-like component of IAT. This mechanistic specificity was impossible in the absence of a time-evolving model of the IAT.

In Study 4 we turned to the topic of learning (and decay thereof) in compatibility effects. A unified model of change in compatibility used a single estimation procedure to show the same qualitative pattern of effects as the original study, while also allowing for the examination of different trial types’ (i.e., compatible vs incompatible) contributions to dynamically-changing compatibility effects. However, it should be noted that, with 2193 participants’ worth of data being fit, the procedure was quite slow. The reported model took approximately 252 h to complete Hamiltonian Monte Carlo sampling with parallel chains, and while the convergence indices met the minimum standard, they were perhaps not as strong as we would like (e.g., one of the random-effects R-hat values was 1.04). We chose not to re-fit the model with more iterations due to time constraints, but we do believe that qualitatively identical results would have been found if we had done so. Regardless, given available Monte Carlo algorithms and the mid-range computational power we utilized (e.g., a desktop computer with 16-core 2.5 GHz processor, sufficient memory, 384 Kb L1d cache, etc.), we suspect that sample sizes of approximately 2200 participants is an upper limit for such analytical techniques at this point in time. Note that approximate (e.g., Variational Bayes) and maximum-likelihood methods may provide similar results with much less computational overhead, and GPU-accelerated methods may provide gains, but a systematic exploration of such methods was outside the scope of the current manuscript.

Another aspect of the current work is its increased attention to the early (historically “practice”) trials of the IAT. The standard IAT d-score algorithm involves weighting those early trials more heavily than the “non-practice” trials; specifically, the scoring algorithm first computes a score for the first 20 trials, then a score for the remaining trials, then averages those scores together, without weighting them differently^14^. This calculation results in a final IAT score that weights the early trials twice as heavily as the later trials. Greenwald et al.^14^ chose this algorithm because its score showed the best correlation with a variety of other indicators; it could be the case that the “improved algorithm” serendipitously tapped into some of the processes revealed by the present work.

Although the patterns described above may be interpreted as learning, as different levels of bias, or bias regulation, it is important to note that changes in behavior need not be intentional or even conscious. Similarly, decreases in bias scores may largely be task learning (e.g., improved narrowing of attentional focus to only the relevant word or face stimulus on a given trial type and a concomitant decrease in the behavioral influence of bias). Such an interpretation would be consistent with Carpenter et al.’s evidence that completing multiple IATs in a sequence increases the reliability of IAT metrics^48^. A task learning account would, however, mean that our observed results would be due to covariance in the population between learning rates and behavioral measures of bias, for which we do not know of any mechanistic basis. A self-regulation account is also consistent with the pattern observed predicting Seeming Racist, in which there was a reliable interaction between asymptotic RT differences and IMS. The results reported here explore patterns of change in the IAT that may have theoretical importance. But the results are not intended to implicate any specific source or process for these patterns of change; further experimentation designed to adjudicate between possible time-dependent components would instead be needed.

Humans are complex, and there are likely multiple constructs and processes interacting to influence observed outcomes in behavior. Previous work has demonstrated that using the traditional, aggregate IAT score as a singular predictor of bias-related behavior may be unwarranted, as “implicit evaluation” interacts with other relevant psychological constructs^16,30^. The present work reveals further complexities — the by-trial response times demonstrate systematic variations and can be used to derived meaningfully distinct metrics. Because our present studies were not set up to distinguish specific mechanisms to account for the various time-dependent metrics, those metrics could relate to numerous processes at play during the IAT, including motor learning, regulatory processes, rehearsal of mental associations^20^, preconscious control^21^, or others. Indeed, the present dissociation of IAT components is orthogonal and compatible with other approaches to dissociating IAT metrics based on theoretical concerns related to prejudice, such as the QUAD model^49^, ReAL model^50^, evidence-accumulation methods (e.g., Drift Diffusion Model^33,34^), and many others^51,52^. To our knowledge, however, none of these established or new approaches include changes over time, thus the present work may fuel additional insights that can interface with these approaches. As statistical technologies increase our ability to estimate more nuanced statistical models, it provides opportunities to develop more complex theoretical models of psychological phenomena.

One reason for the ubiquity of the IAT is that, in contrast to early studies on automatic race bias^23^, the present-day IAT is relatively easy to administer and access data. Such accessibility has led to many researchers, or ad hoc researchers, using it without a rigorous understanding of the IAT, its underlying assumptions, or the intricacies of RT psychometrics. Someone with no training in the cognitive sciences can administer an IAT, which yields a simple numerical score that they can ostensibly interpret. In this way, the IAT grants a “sheen” of methodological sophistication to studies that might otherwise fall flat. This phenomenon — of a measure granting perceived sophistication to otherwise potentially underwhelming research — warrants greater attention, as psychological scientists work to maintain a rigorous, cumulative science.

To be clear, our goal in this discussion is not to criticize the IAT in general, or the many scientists who use it rigorously in their research. Our “criticism”, if any, is centered on the oversimplification of the IAT and its interpretations, and its pervasive use by researchers or laypeople who lack sophisticated training in the domain of cognitive RT tasks. The present work reveals potential complexities within people’s performance on the IAT, which we hope will be generative and useful for those who use the IAT as part of a program of rigorous, theory-driven research.

### Supplementary Information

Supplementary Information.

## Data Availability

The data from Study 1 are available with. 10.5334/jopd.ac; The data from Studies 2 and 3 are available at zenodo.org/record/6,454,417; The data from Study 4 is available at osf.io/serq4/.
