# Supplementary material for: Robust within-session modulations of IAT scores may reveal novel dynamics of rapid change
Source: Sci Rep. 2023 Sep 27;13:16247. doi: 10.1038/s41598-023-43370-w (PMC10533519; doi:10.1038/s41598-023-43370-w)
Supplement: Supplementary file 1 — Supplementary Information. [file 41598_2023_43370_MOESM1_ESM.docx]

# Supplemental Information

## Model specification (Study 2)

The nonlinear generalized mixed-effects model was fit using the R package brms ^35^. Each model was run on 4 sampling chains, each with 10,000 iterations, with the first 6,000 iterations discarded as warmup. A thinning interval of 2 was used. All priors were default or were extremely wide (i.e., outside the plausible range of RT) in order to maintain data-driven interpretations of fit parameters. Each nonlinear parameter was estimated on a log scale. Model convergence was indicated by all R-hat values being below 1.01, and all Bulk and Tail Effective Sample Sizes being above 1,000.

Models in Study 2 and Study 3 used an ex-Gaussian response distribution due to the outcome variable being response times. By default ex-Gaussian models in **brms** involve predicted changes in the Gaussian mean component of the ex-Gaussian distribution. However, due to the exponential component’s ability to parsimoniously capture changes in mean RT in conjunction with RT variance, models reported here predict variation in the exponential component. More precisely, in **brms** the default predicted value (i.e., main formula left-hand-side) reflects shifts in the mean of the Gaussian component added to the predicted exponential component. As such, the model specification described below was paralleled between the first line (the predicted value of the exponential component, our value of interest, plus an additive offset for the Gaussian component) and the second line (the same as the first line, but without the Gaussian component). The Gaussian mean and variance components were each estimated using by-participant random intercepts, with by-participants random slopes for a stimulus being a word vs. a face (i.e., to account for by-participant differences in reading speed). These details can be seen in the model formula.

Model Formula:

| latency ~ exp(noiseMean) + exp(rtAsym) + (exp(rtStart) - exp(rtAsym)) * (2^((1 - trialNum)/(2 + 2^rtRate))) |
| --- |
| beta ~ exp(rtAsym) + (exp(rtStart) - exp(rtAsym)) * (2^((1 - trialNum)/(2 + 2^rtRate))) |
| rtStart ~ incFirst * inc + (inc \|\| subject) |
| rtRate ~ incFirst * inc + (inc \|\| subject) |
| rtAsym ~ incFirst * inc + (inc \|\| subject) |
| noiseMean ~ (isWord \|\| subject) |
| sigma ~ (1 \|\| subject)  *Naming conventions: latency = Response Time; noiseMean = mean of Gaussian component; rtAsym = asymptote of exponential component; rtStart = starting point of exponential component; trialNum = trial number (1 to 60); rtRate = log time constant of change in exponential component; beta = expected value of exponential component; incFirst = “incompatible” block first (+.5) or second (-.5); inc = whether the block was “incompatible” (+.5) or “compatible” (-.5); subject = participant ID; isWord = whether or not the trial’s stimulus was a word (+.5) or an image (-.5); sigma = standard deviation of the Gaussian component.* |

Model Formula (null model; constant over time):

| latency ~ noiseMean + exp(expoComp)  expoComp ~ incFirst * inc + (inc \|\| subject)  noiseMean ~ (is_word \|\| subject)  sigma ~ (1 \|\| subject)  beta ~ exp(expoComp) |
| --- |

*Naming conventions: see above*

## Model specification (Study 3)

As in Study 2, nonlinear generalized mixed-effects models were fit using the R package **brms** (Buerkner, 2017). Each of the time-evolving parameters (*rtStart, rtRate,* and *rtAsym*) were themselves the functions of simultaneously-estimated linear mixed-effects models with the fixed main effects of IAT type (*expGroup*, good/bad or competent/incompetent; zero-centered), trial type (zero-centered), and their interaction, as well as the by-participant random effects that paralleled the fixed effects.

Each model was run on 4 sampling chains, each with 10,000 iterations, with the first 6,000 iterations discarded as warmup. A thinning interval of 2 was used. All priors were default or were extremely wide (i.e., outside the plausible range of RT) in order to maintain data-driven interpretations of fit parameters. Each nonlinear parameter was estimated on a log scale. Model convergence was indicated by all R-hat values being below 1.01, and all Bulk and Tail Effective Sample Sizes being above 1,000.

Model Formula

| latency ~ exp(noiseMean) + exp(rtAsym) + (exp(rtStart) - exp(rtAsym)) * (2^((1 - trialNum)/(2 + 2^rtRate))) |
| --- |
| beta ~ exp(rtAsym) + (exp(rtStart) - exp(rtAsym)) * (2^((1 - trialNum)/(2 + 2^rtRate))) |
| rtStart ~ expGroup * inc + (expGroup * inc \|\| subject) |
| rtRate ~ expGroup * inc + (expGroup * inc \|\| subject) |
| rtAsym ~ expGroup * inc + (expGroup * inc \|\| subject) |
| noiseMean ~ (isWord \|\| subject) |
| sigma ~ (1 \|\| subject)  *Naming conventions: see Model Specification (Study 2)* |
|  |

Model Formula (null model; constant over time):

| latency ~ noiseMean + exp(expoComp)  expoComp ~ expGroup * isInc + (expGroup * isInc \|\| subID)  noiseMean ~ (isWord \|\| subID)  sigma ~ (1 \|\| subID)  beta ~ exp(expoComp) |
| --- |
| *Naming conventions: see Model Specification (Study 2)* |

## Model specification (Study 4)

The Study 4 model was specified similarly to Studies 2 and 3, concerning the software, distribution, sampling parameters, and so on.

Model formula

latency ~ pAsym + ((pStart) - (pAsym)) * 2^((1 - blockTrialNum)/(2^(pRate))) + meanOffset

pStartXform ~ learnCond * isInc + genderC + ageC + (isInc || sessionID) + (learnCond * isInc + genderC + ageC || study)

pRate ~ learnCond * isInc + genderC + ageC + (isInc || sessionID) + (learnCond * isInc + genderC + ageC || study)

pAsymXform ~ learnCond * isInc + genderC + ageC + (isInc || sessionID) + (learnCond * isInc + genderC + ageC || study)

meanOffset ~ (1 | sessionID) + (1 | study)

pStart ~ exp(pStartXform)

pAsym ~ exp(pAsymXform)

beta ~ pAsym + ((pStart) - (pAsym)) * 2^((1 - blockTrialNum)/(2^(pRate)))

*Naming conventions: pAsym, pStart, and pRate indicate the parameters of exponential change. meanOffset is the mean of the Gaussian component of the ex-Gaussian distribution. learnCond is the learning condition, with the control condition as the reference level. isInc is the compatibility variable (coded as -.5 compatible, +.5 incompatible), in the direction of the baseline tendency of participants (i.e., the direction expected in the control condition; the opposite direction as expected in the learning conditions). genderC and ageC are zero-centered gender and age, respectively. sessionID is participant ID. study is the study ID (from studies 3a-e, Kurdi & Banaji, 2019).*

________


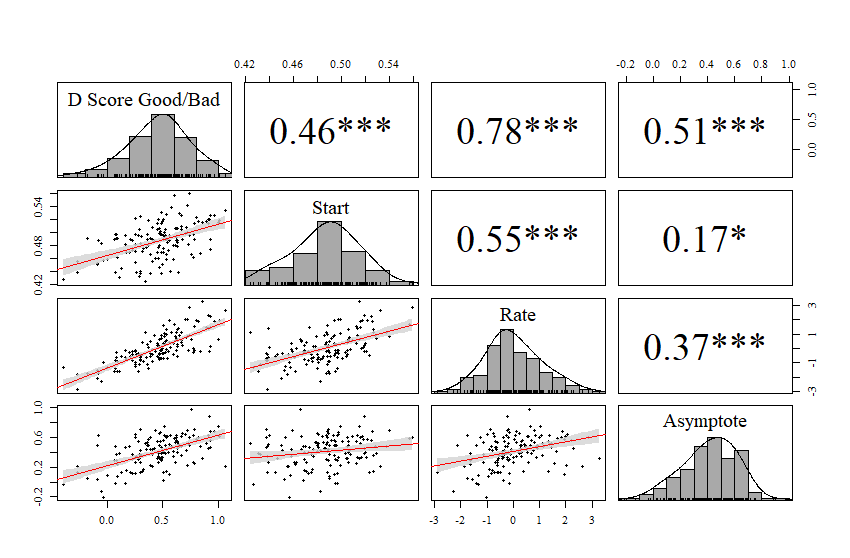


*Fig S1. Rank correlations between participant-level estimates of indices of Race IAT (Study 3). Stars indicate *p<.05, **p<.01, ***p<.001.*
